# Examining Learning Experience and Satisfaction of Accounting Students in Higher Education before and amid COVID-19

**DOI:** 10.3390/ijerph192316164

**Published:** 2022-12-02

**Authors:** Mohamed A. Shabeeb, Abu Elnasr E. Sobaih, Ibrahim A. Elshaer

**Affiliations:** 1 Department of Accounting, School of Business, King Faisal University, Al-Ahsa 31982, Saudi Arabia; 2 Accounting Department, Faculty of Commerce, South Valley University, Qena 1464040, Egypt; 3 Management Department, College of Business Administration, King Faisal University, Al-Ahsa 31982, Saudi Arabia; 4 Hotel Management Department, Faculty of Tourism and Hotel Management, Helwan University, Cairo 12612, Egypt; 5 Hotel Studies Department, Faculty of Tourism and Hotels, Suez Canal University, Ismailia 41522, Egypt

**Keywords:** students’ satisfaction, learning experience, electronic learning experience, COVID-19 pandemic, accounting students

## Abstract

The widespread outbreak of the COVID-19 virus had substantial impacts on higher education, which turned into distance using virtual environments and electronic (e) learning platforms. There is a growing body of research on the effect of COVID-19 on students’ education and e-learning experiences amid the pandemic. However, limited research was performed to assess the learning experience before and amid the COVID-19 pandemic among students in specific disciplines such as accounting. The current research compares accounting students’ learning experience and satisfaction before and amid the COVID-19 pandemic. We distributed a pre-tested questionnaire online to students through our colleagues. The results of the SEM multi-group analysis with Amos indicated significant differences between students’ experience before and amid the COVID-19 pandemic, which had a significant influence on their satisfaction. Accounting students were found to have more participation in learning, to receive proper support and motivation, and to have better assessment and feedback before than amid COVID-19. However, they had better access to information and learning resources and were able to construct knowledge amid the pandemic using e-learning than before the pandemic. Several implications from the findings are raised and discussed.

## 1. Introduction

Amid the announcement by WHO “World Health Organisation”, at the beginning of 2020, that COVID-19 was a worldwide pandemic, policymakers in most countries universally shifted their educational system to the distance. This was undertaken to ensure the protection of students because of the widespread coronavirus [1]. Electronic (e) learning was the norm and the sole tool of learning in most universities for at least a couple of semesters, thanks to the leaders of universities, who were also keen to protect their students and staff while maintaining the quality of education. Universities provided their staff and students with every possible tool to enhance their e-learning experience of students with a virtual learning environment [1].

As expected, scholars responded quickly to the pandemic and its influence on students and their learning experience. A growing academic body of research was conducted to understand the impacts of COVID-19 on education and learning (e.g., [2,3,4]); to highlight the challenges and/or opportunities facing e-learning amid the COVID-19 pandemic (e.g., [5,6]); to stress the value of e-learning using either a formal learning system or social network sites (e.g., [7,8,9,10,11]); to maintain academic performance using e-learning (e.g., [12,13]); to enhance student satisfaction and engagement (e.g., [14,15]); to create positive e-learning experiences (e.g., [16,17]; and to understand education post-COVID-19 pandemic (e.g., [18]). 

Learning experiences before COVID-19 were mostly based on face-to-face interaction between students and their educators; however, learning amid COVID-19 turned virtual, where the interaction is online using various digital platforms [1]. There is some important questions about students’ learning experiences amid COVID-19, which could influence the creation of positive learning experiences as well as students’ satisfaction post-COVID-19 pandemic. The research questions are: To what extent do students’ satisfaction with learning experiences differ amid the COVID-19 pandemic in contrast to before? What are the factors that have the most influence on student learning experience and satisfaction before and amid the pandemic caused by COVID-19? What are the lessons we could learn from the COVID-19 pandemic?

A review of research to date on students learning experiences, engagement, and satisfaction amid COVID-19 showed that most research examined the impact of COVID-19 on teaching and learning and students’ perception of e-learning in general (e.g., [3,4,16]). Similarly, there was an increasing body of research examining the e-learning experience of students, especially medical and nursing students (e.g., [19,20,21,22,23]). These studies highlighted the limitation of e-learning in the interaction between students and educators, especially in practical courses and training sessions that required physical attendance. However, limited research was undertaken to compare students learning experiences before and amid COVID-19 in many disciplines, such as accounting. There was an attempt pre-COVID-19 pandemic to compare distance and traditional learning among accounting students [24]. It was found that distance learning is a supporting tool beside traditional learning, which is face-to-face, but it cannot replace it. However, students amid COVID-19 adopted distance learning as a single learning tool, which requires an examination, as was undertaken by the current study.

Prior to the coronavirus outbreak, the majority of universities in Saudi Arabia, such as in many other countries, did not embrace online learning technologies in education. However, this sudden adoption of an online learning platform in the business and accounting departments could be detrimental to the outcomes of the educational process [25]. There might be an issue with the negative consequences, especially the quality of outcomes among accounting students. One important issue that may lead to the inappropriate performance of accounting students during the online learning process compared to face-to-face education is its quantitative nature, with many tables, figures, and numbers [26]. Studies, e.g., [27,28], reported that quantitative online course results were poorer than those of qualitative courses. The quantitative nature of accounting courses requires a high level of interaction between instructors and students. Ref. [29] found that less communication levels between instructors and students is one of the main problems that face online courses. Additionally, accounting curricula rely heavily on technology, and numerous technologies are applicable to these curricula [30,31,32]. Most of these technologies may require software that cannot be available outside the university’s campus and require more interaction when discussed. According to Means et al. [33], online education approaches are viewed as less effective than conventional teaching, which is face-to-face and in universities, e.g., accounting education. However, if accounting education is severely affected by COVID-19, this will detrimentally affect the profession and the production of skillful accountants for the industry [34].

The current research aimed to compare accounting students’ learning experiences and satisfaction before and amid the COVID-19 pandemic. The research examined the factors that create positive learning experiences before and amid the COVID-19 pandemic. It examined the influence of e-learning experiences on student satisfaction before and amid the COVID-19 pandemic. It is anticipated that the results of this research will support the provision of positive learning experiences and enhance the e-learning experience post-COVID-19 pandemic. Therefore, in the next sections of the paper, we build the relationship between the learning experience and student satisfaction. We then examine this relationship with accounting students before and amid the COVID-19 pandemic. Using multi-group analysis, we compare student learning experiences and satisfaction before and amid the COVID-19 pandemic. We then discuss these results and conclude the research. 

## 2. Literature Review

### 2.1. Learning Experiences in Higher Education before and amid COVID-19

As highlighted earlier, learning amid COVID-19 turned into distance learning using digital platforms. There were some attempts by scholars to assess the efficiency of e-learning compared to a traditional classroom, especially among medical and health students. The recent study of Nalini et al. [35] adopted a paired sample test to the comparison between the pre- and post-test of two different groups (online and traditional), and the results showed statistically significant differences between online and traditional, where online learning was found to be better for encouraging deeper and independent learning. The study of Anwar et al. [19], who tested the medical and dental students’ e-learning experiences in private education institutions, showed that they were prepared to make the transition to online learning because they found their experience with e-learning positive. However, they called for more studies to address the deficiencies of this shift on the quality of learning outcomes. Another research paper conducted by Nepal [20], which was implemented on nursing students, showed that nursing students had a positive attitude towards e-learning amid the pandemic. However, students reported internet problems and technological issues, but if the obstacles are controlled, then e-learning can be an alternative tool to traditional learning. A study on Jordanian medical students showed that e-learning had several technical and infrastructure obstacles that affected the learning experience and satisfaction of students [21]. An interesting study by Kaur et al. [22] compared traditional forms of learning to e-learning during the COVID-19 pandemic on medical undergraduate students. The results showed that e-learning was less effective compared to traditional classroom learning. They found that, while e-learning is as equally effective as conventional learning in communication and students building skills, it is not of the same interaction level and is suitable for practical courses. They recommended that e-learning should be adopted as a supporting learning tool and not as a substitute for traditional learning. In accounting education context, the limited published studies (e.g., [25]) show that this sudden shift in accounting education towards e-learning could negatively affect the learning outcomes. This is because the quantitative nature of accounting education with many tables, figures, and numbers [26] requires a high level of interaction between the instructor and students [29]. 

### 2.2. Student Learning Experiences and Satisfaction in Higher Education

Student satisfaction with teaching methods and the learning process is crucial for the sustainability of higher education [11,12]. Hence, leaders of higher education institutions pay much attention to the creation of a positive experience that achieves student satisfaction [16]. The antecedents of student learning experiences vary; however, the model of student learning experience suggested by Awidi [36] was examined in several studies. According to Awidi [36], the learning experience has six antecedents: knowledge construction and personal reflection, feedback, assessment, motivation and support, access to resources and information, and participation and collaboration. Awidi et al. [37], Prakash and Saini [38], and Alyahya [39] scales are appropriate for examining student experiences and their relationship with their satisfaction with learning. Awidi [36] argued that these are the determinants of learning experiences and have a positive influence on student satisfaction. In accounting, students require more interaction with their instructors about statistics and numbers; however, there were less communication levels between instructors and accounting students amid COVID-19 [34]. Hence, it is expected there are differences between accounting students before COVID-19, which used traditional face-to-face learning, and amid COVID-19, which became e-learning. Relying on these arguments, we could assume that:

**Hypothesis** **1.** *A significant difference is expected in the relationship between experience with knowledge construction and student satisfaction before and amid COVID-19.*

**Hypothesis** **2.** *A significant difference is expected in the relationship between experience with feedback and student satisfaction before and amid COVID-19.*

**Hypothesis** **3.** *A significant difference is expected in the relationship between experience with assessment and student satisfaction before and amid COVID-19.*

**Hypothesis** **4.** *A significant difference is expected in the relationship between experience with support and motivation and student satisfaction before and amid COVID-19.*

**Hypothesis** **5.** *A significant difference is expected in the relationship between experience with Access to knowledge and student satisfaction before and amid COVID-19.*

**Hypothesis** **6.** *A significant difference is expected in the relationship between experience with participation and student satisfaction before and amid COVID-19.*

## 3. Methods 

### 3.1. Targeted Sample

Students majoring in accounting at the School of Business (SoB) in public higher education institutions in Saudi Arabia (KSA) were the focus of this study. These universities were among those that relied heavily on face-to-face lectures before the COVID-19 pandemic and were pushed to transfer to online platforms throughout the pandemic in order to continue giving lectures and maintaining contact with students.

The research team circulated the developed questionnaire to the targeted accounting students via personal relationships and networks. They were requested to distribute and share the survey link via WhatsApp or email. Students were allowed to either answer the anonymous survey or choose not to. To make sure that no one else could reply to the questionnaire, the students were required to write their formal email address before completing the questionnaire. To ensure that no one else could reply to the questionnaire, the students had to write their formal university email addresses before completing the questionnaire.

Participation in the survey was entirely voluntary, and anonymity was preserved to protect respondents’ privacy; all information that could be used to identify participants’ identity was deleted from the results. Student name, age group, and institution name were not obligatory questions. A total of 530 questionnaires were distributed, 500 valid questionnaires were retained with no missing data, and there was a 94% response rate. The questionnaire was distributed in September and October 2021. The targeted students were asked to evaluate the same questions before and during the COVID-19 pandemic.

### 3.2. Instrument and Scale Development

A multi-item scale (5-point Likert scale) was applied to assess the research dimensions. The study scale consisted of seven factors. Six of them were adopted from Awidi [37] in order to assess learning experiences before and during the COVID-19 pandemic. The learning experience has six latent dimensions with 26 items (questions). These latent dimensions are as follows: “critical reflection and knowledge construction” (CRAKS), “feedback” (FEED), “assessment” (ASSES), “participation and collaboration” (PAC), “support and motivation”, (SAM), “access to information and learning resources” (AIR), while the student satisfaction (SATIS) measure was adopted from Jiang et al. [40] and has four items (i.e., “Overall, I am satisfied with the ease of completing my tasks by using the online learning platforms”). 

The online questionnaire was structured and designed to match the recommendations illustrated in the previous literature [41]. After generating the scale items, one researcher converted the questionnaire into an online version that was thoroughly reviewed by the research team before the distribution of the URL to the targeted students. The study’s main aims were well defined, and the targeted accounting students were requested to contribute to answering the survey. Accounting students (study sample) were knowledgeable of their confidentiality and anonymity. Students obtained the URL of the questionnaire (in English and Arabic) via social media profiles or university emails. The research members followed up on the replies on a daily basis. Participant personal information was optional (student name, student phone number, email address, and social media profiles) and was located at the bottom of the questionnaire.

After the scale was translated from English to Arabic, 17 accounting students and 16 accounting professors were asked to evaluate its clarity, simplicity, and suitability. Throughout this process, no significant changes were made; however, a few suggestions for language clarity were implemented. For the purpose of determining the reliability of the scale items, Cronbach’s alpha (*a*) values were assessed. The alpha (*a*) scores ranged from 0.91 to 0.96, which is higher than the recommended cut-off value of 0.7 that Nunnally [42] recommended.

Several procedures were executed to detect common method variation (CMV) in the self-reported online survey data [43]. For example, (1) the dependent variable (student satisfaction) was allocated in the survey to be before the dependent variables (learning experience dimensions). (2) The identities and confidentiality of respondents were secured. (3) We employed Harman’s single-factor method, where all the survey items were subjected to exploratory factor analysis (EFA) in the SPSS software with the limitation that only one factor should be retrieved without rotating the data. The findings showed that CMV was not an issue at any point during our investigation because just one variable accounted for a variance of 37% [44].

### 3.3. Methods of Data Analysis

In our study, two main data analysis methods were employed. First, descriptive analysis (respondent demographics, means (*M*), and standard deviation (S.D)) was conducted. Second, two multivariate data analysis (MVA) techniques were employed, which were (1) confirmatory factor analysis (CFA) and (2) structural equation modeling (SEM). SEM was preferred as the main data analysis approach because it can concurrently test and assess complicated latent multidimensional hypotheses. SEM can assess complicated relationships while taking into account the possibility of measurement error [43]. Several SEM goodness of fit (GOF) criteria were employed as follows: “2/df, RMSEA, SRMR, CFI, TLI, NFI, PNFI, and PCFI”, as suggested by various sources [45,46,47,48]. SPSS 25 and AMOS 24 were used for data analysis.

## 4. Data Results 

### 4.1. Descriptive Analysis Results

There was nearly an equal distribution of the study respondents between males (53%) and females (47%). The mainstream accounting students, as expected, were under 26 years old (92%). The participants’ responses were in the form of a number between 1 and 5, with 5 denoting “strongly agree” and 1 denoting “strongly disagree”. The range of values for the mean was from 3.52 to 4.15, and the range of values for the standard deviation was from 0.914 to 1. 292. As a direct consequence of this, the data were spread out more evenly and were not as concentrated in the center [47]. Furthermore, the analyses of the skewness and kurtosis ranges demonstrated that there were no values that were greater than −2 or +2, indicating that the data were normalized using a univariate approach [46].

### 4.2. Multivariate Analysis Results

We conducted a two-phase successive structural equation modeling (SEM) approach as recommended by [48]. In phase 1, the validity and reliability of the scale were evaluated with a first-order CFA model (measurement) using AMOS v24 and a maximum likelihood estimation procedure. In phase 2, the nomological model (structural) was evaluated using the same procedure to test the study hypotheses. Furthermore, a multi-group analysis method was conducted in Amos vs24 to detect if the tested hypotheses differed before and amid the COVID-19 pandemic.

### 4.3. Phase 1: CFA Models (Construct Validity and Reliability)

Two first-order CFA models (before and amid the COVID-19 pandemic) were drawn and run in Amos v24 to evaluate the discriminant and convergent validity of the employed scale (seven latent dimensions and 30 variables). The GoF criteria showed a good fit in the two models as displayed in Table 1.

The seven dimensions’ composite reliability (C.R) values in the two models (see Table 1) showed good internal consistency because they ranged from 0.92 to 0.97 and consequently exceeded the suggested threshold score of 0.70 [39]. Moreover, the dimensions’ reliability was supported by assessing the Cronbach’s alpha scores, which all were found to be higher than the required threshold point of 0.70, as depicted in Table 1 [42]. Additionally, the results in Table 1 further support the scale convergent validity, as all standardized factor loadings (SFL) were found to be significant with high loadings (ranging from 0.78 to 0.98 in the two models). The average variance extracted (AVE) for all the seven employed dimensions was found to be higher than 0.50, as recommended by Hair et al. [46], which further supports the convergent validity of the employed scale. Furthermore, as seen in Table 1, all maximum shared variance (MSV) scores were found to be lower than the corresponding AVE scores, demonstrating proper discriminant validity [45]. Finally, as shown in Table 2, the squared root of the AVE values (bold values) was higher than the intercorrelation of the variables (below bold variables), giving more evidence that supports that the scale has adequate discriminate validity [44].

### 4.4. Phase 2: Hypotheses Testing in the Structural Models

Following the review of the relevant prior research, a particular theoretical model was justified. Subsequently, primary data were gathered and examined in order to ascertain whether or not they matched the assumed theoretical model [45]. Depending on how well the assumed model fit the data, it was either disapproved or approved.

**Figure 1:**
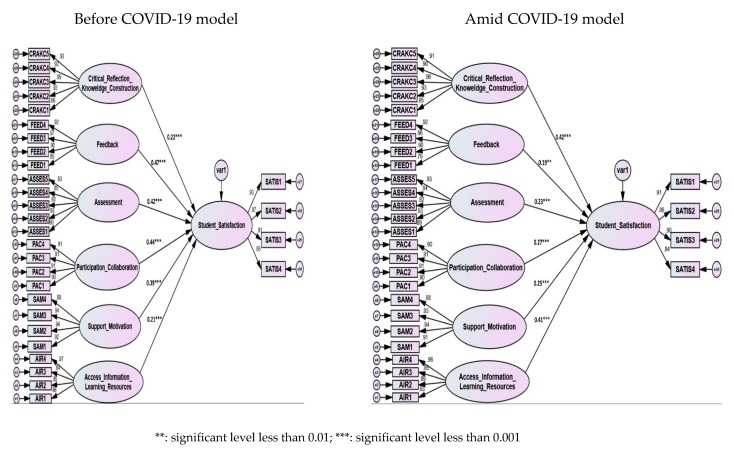
The structural results for the two comparative models (before and amid COVID-19).

Table 3 and Figure 1 show the GoF for the two proposed and tested structural models (before and amid the pandemic caused by COVID-19). The chi-square GoF analysis was significant (*p* less than 0.01) in both of the models that were put to the test, which suggests that the null hypothesis (models fit the data well) was not accepted. That is to say, the real covariance matrix, denoted by the letter S, did not match the covariance matrix that was calculated, denoted by the symbol (∑k). However, because the size of the sample affected the *p* value, and the value itself was always significant, other several GoF were considered, such as “Standardized Root Mean Squared” (SRMR), “Root Mean-Square Error Approximation” (RMSEA), “normed chi-square” (chi-square divided by degree of freedom), “Comparative Fit Index” (CFI), “Tucker Lewis index” (TLI), and Parsimony Comparative Fit (PNFI) [43,45]. As depicted in Table 2, Model 1 (before the COVID-19 pandemic) demonstrated somewhat better GoF criteria than Model 2 (amid the COVID-19 pandemic). Generally, the two models had an adequate fit for data. All the paths’ coefficients (hypotheses) in the two models were found to be significant and positive as shown in Figure 1 and Table 3. 

### 4.5. Multi-Group Analysis Results 

To test if the relationships between learning experience dimensions and student satisfaction variables differed before the COVID-19 pandemic (Model 1) and amid the pandemic (Model 2), the two groups of data were compared in order to determine whether or not there are any deviations in the model path coefficients (i.e., variance). An SEM multi-group analysis technique was employed with Amos program version 24. The two models were compared in order to identify any deviations in the model paths (i.e., variant). An examination of the differences between the full structural models of the two groups under study could be conducted through the use of a chi-square (χ^2^) difference analysis. Comparing the estimated chi-square value of the free unconstrained (baseline) model and the fixed constrained (structural weights) model disclosed a significant difference with a *p* value less than 0.001 between the two tested models. Consequently, the results suggest that one/or more of the path coefficients between the two tested models was not equivalent [47].

As can be seen in Table 3 and pictured in Figure 1, all the path coefficients s were found to be positive and significant in the two evaluated models. Nevertheless, the Amos results indicated that the GoF and most of the regression weights of Model 1 were found to be higher than the GoF and the same regression weights in Model 2. More specifically, the impact of feedback (FEED) on student satisfaction in Model 1 (before the pandemic) was shown to have a greater positive effect and a more significant value (β = 0.47, *p* <0.001) (H2) than in Model 2 (β = 0.19, *p* < 0.01). Similarly, ASSESS was found to have a higher positive (β = 0.42) significant effect on student satisfaction (H3) in Model 1 than the situation in Model 2 (β = 0.23).

The impact of participation and collaboration (PAC) on students’ satisfaction (H4) in Model 1 (β = 0.44, *p* < 0.001) was higher than that in Model 2 (β = 0.27, *p* < 0.001). Likewise, support and motivation (SAM) were found to have a higher significant positive effect (β = 0.39, *p* < 0.001) on students’ satisfaction (H5) in Model 1 than in Model 2 (β = 0.25, *p* < 0.001).

On the other hand, during the pandemic (Model 2), critical reflection and knowledge construction were found to have a higher and more positive significant impact on students’ satisfaction (β = 0.42, *p* < 0.001) than before (Model 1) the pandemic (β = 0.22, *p* < 0.001), supporting H1. Similarly, the association between access to resources and information and satisfaction was found to be higher amid the pandemic (β = 0.41, *p* < 0.001) than before the pandemic (β = 0.21, *p* < 0.001), supporting H6. 

Table 3 also demonstrates that the explanatory power (squared multiple correlations) of Model 1 was higher (0.83) than that of Model 2 (0.65). Therefore, Model 1 (before the COVID-19 pandemic) showed a higher explanatory power than model 2 (amid the pandemic) in explaining students’ satisfaction with the learning process. 

## 5. Discussion 

We undertook this study to examine whether there are significant differences in learning experiences and its relationships with accounting student satisfaction, both before and amid COVID-19. Accounting students used to have traditional learning in face-to-face classrooms with little (as supplement) or no e-learning before COVID-19; however, amid COVID-19, they only have access to e-learning. Hence, there was concern about the detrimental effect of COVID-19 on accounting education and the quality of graduates in terms of knowledge and skills [34]. This is because accounting education requires more interactivity with instructors, which may be limited in e-learning compared to face-to-face [25]. In this study, we examined whether this shift in learning affected the student learning experience and their satisfaction. The results of our research showed an overall positive relationship between learning experience and satisfaction, before COVID-19 (model 1) and amid the COVID-19 (model 2) pandemic. We found that the influence of both assessment and feedback on student satisfaction before COVID-19 was shown to have a greater positive effect and a more significant value than amid the pandemic. This is because students in the accounting discipline would like to receive in-person assessments and obtain in person feedback; the quantitative nature of this discipline with lots of numbers requires personal feedback and assessment. Therefore, students stated that they found their learning experience with both feedback and assessment better in traditional learning (before COVID-19) than e-learning (amid COVID-19). Despite the fact that students may find it easier to submit their assignments for assessment and undertake exams online [36], a traditional classroom is more effective in assessment and feedback than learning [35]. 

The results also showed that the impact of students’ participation and collaboration as well as support and motivation on their satisfaction was higher with traditional classroom learning (i.e., before COVID-19) than in e-learning (amid the COVID-19 pandemic). Students found themselves more supported, motivated, engaged, and more likely to participate in the course activities in a traditional classroom than in e-learning. These findings are in line with previous literature review [25,26,29], such as that accounting education requires more participation and engagement of students during the discussion to ensure they sufficiently understood the information given with many tables and numbers. Hence, these issues were higher before the pandemic with face-to-face interaction than after the pandemic, since e-learning was a single learning tool. 

On the other hand, we found that access to information and resources was higher amid the pandemic than before the pandemic. Students found themselves having more access to information online relevant to their courses at their convenience. They can access this information online anytime they want. It was appropriate for them that the learning sources and information is available all the time and they can access it as much as they want. This supports the findings of Alyahya et al., [39], who found that learning facilitates access to information and learning and, hence, creates a positive learning experience. This has a more positive significant influence on students’ satisfaction than before the pandemic. Interestingly, students have better personal reflection and knowledge construction because of e-learning amid the pandemic than before the pandemic. They found that e-learning gave them more confidence to explore course content and solve a problem, which supports the findings of Elshaer and Sobaih [16] and Alyahya et al. [39]. The results overall showed that the influence of experience with traditional learning has a higher effect on students’ satisfaction than the e-learning experience.

Our findings are of significant value for accounting education (and other similar disciplines). Our research showed that traditional learning (before the pandemic) is more appropriate than e-learning (amid the pandemic) for creating more support, motivation, collaboration, and participation for students in the e-learning process due to the quantitative nature of accounting education. Additionally, face-to face teaching was found to be more useful for giving assessment and feedback to students than e-learning. Hence, students were more satisfied with their traditional classroom experience than e-learning in these issues. Nonetheless, they found e-learning more appropriate than traditional learning for accessing information and resources, as well as knowledge construction and personal reflection. These findings confirmed that a blend of traditional and e-learning post-pandemic would be more appropriate for creating a learning experience and enhancing students’ satisfaction, which definitely influences their academic performance [12]. 

## 6. Conclusions

The current study compared the learning experiences and satisfaction of accounting students before and amid COVID-19 for ensuring a better and quality education. The results showed an overall positive relationship between learning experience and satisfaction, before (model 1) and amid COVID-19 (model 2). The results confirmed more positive experiences with feedback, assessment, support and motivation, and participation and collaboration before COVID-19 than amid COVID-19. This means that there were more positive experiences with traditional learning than e-learning in relation to these four factors. However, there were more positive experiences with knowledge construction and personal reflection, as well as access to information and resources amid COVID-19 than before COVID-19. This reflects that e-learning, which was provided amid COVID-19, supported students to have access to information and resources and enhance their personal reflection and knowledge construction. These results acknowledge blended learning post-COVID-19 education to gain the benefits of different types of learning and enhance students’ satisfaction, as well as their academic performance. 

## Figures and Tables

**Table 1:** Psychometric properties of the two tested models.

		Model 1: Before COVID-19					Model 2: Amid COVID-19				
Factors	Variables Abbreviations	SFL	α	C.R	AVE	MSV	SFL	α	C.R	AVE	MSV
**Student satisfaction** **(SATIS)**	SATIS1	0.922	0.92	0.93	0.78	0.62	0.905	0.93	0.92	0.76	0.47
	SATIS2	0.868					0.858				
	SATIS3	0.904					0.897				
	SATIS4	0.848					0.834				
**Critical reflection and knowledge construction ** (**CRAKC**)	CRAKC1	0.959	0.91	0.97	0.87	0.38	0.953	0.92	0.97	0.87	0.35
	CRAKC2	0.934					0.930				
	CRAKC3	0.947					0.962				
	CRAKC4	0.917					0.906				
	CRAKC5	0.931					0.917				
**Feedback** **(FEED)**	FEED1	0.845	0.96	0.95	0.82	0.15	0.784	0.95	0.94	0.79	0.14
	FEED2	0.908					0.906				
	FEED3	0.965					0.954				
	FEED4	0.921					0.917				
**Assessment** **(ASSES)**	ASSES1	0.908	0.94	0.96	0.83	0.47	0.893	0.93	0.95	0.81	0.47
	ASSES2	0.949					0.931				
	ASSES3	0.930					0.922				
	ASSES4	0.849					0.845				
	ASSES5	0.931					0.926				
**Participation and collaboration** (**PAC**)	PAC1	0.918	0.95	0.94	0.82	0.38	0.913	0.93	0.94	0.82	0.35
	PAC2	0.906					0.900				
	PAC3	0.902					0.899				
	PAC4	0.903					0.897				
**Support and motivation** (**SAM**)	SAM1	0.914	0.94	0.95	0.84	0.62	0.911	0.93	0.95	0.83	0.47
	SAM2	0.943					0.937				
	SAM3	0.937					0.928				
	SAM4	0.880					0.883				
**Access to information and learning resources ** (**AIR**)	AIR1	0.962	0.93	0.94	0.82	0.47	0.928	0.92	0.95	0.81	0.46
	AIR2	0.848					0.886				
	AIR3	0.840					0.839				
	AIR4	0.973					0.959				

Model 1 CFA: “χ^2^ (384, N = 500) = 804.48, *p* < 0.001, normed χ^2^ = 2.095, SRMR = 0.011, RMSEA = 0.021, CFI = 0.986, NFI = 0.970, TLI = 0.971, PNFI = 0.761, and PCFI = 0.781)”. Model 2 CFA: “χ^2^ (384, N = 500) = 847.488, *p* < 0.001, normed χ^2^ = 2.207, SRMR = 0.021, RMSEA = 0.031, CFI = 0.988, NFI = 0.978, TLI = 0.982, PNFI = 0.775, and PCFI = 0.790)”. “Note: SFL = standardized factor loading; a = alpha value; C.R = composite reliability; MSV = maximum shared variance; AVE = average variance extracted”.

**Table 2:** Validity results.

Before COVID-19 Model								Amid COVID-19 Model						
	AIR	FEED	CRAKC	SATIS	ASSES	PAC	SAM	AIR	FEED	CRAKC	SATIS	ASSES	PAC	SAM
**AIR**	0.908 *							**0.904**						
**FEED**	0.347	**0.911**						0.338	**0.893**					
**CRAKC**	0.331	0.335	**0.938**					0.313	0.331	**0.934**				
**SATIS**	0.398	0.128	0.239	**0.886**				0.376	0.118	0.236	**0.874**			
**ASSES**	0.691	0.317	0.329	0.304	**0.914**			0.688	0.309	0.320	0.294	**0.904**		
**PAC**	0.330	0.387	0.620	0.132	0.468	**0.907**		0.314	0.376	0.595	0.110	0.458	**0.902**	
**SAM**	0.491	0.097	0.142	0.792	0.356	0.025	**0.919**	0.470	0.082	0.135	0.689	0.349	−0.001	**0.915**

* Bold values are the squared root of the AVE values.

**Table 3:** Hypotheses results for the two comparative models (before and amid COVID-19 models).

		Before COVID-19			Amid COVID_19		
Tested Relationships		Β-Value	SMC	Results	Β-Value	SMC	Results
H1	Critical reflection and knowledge construction → SATIS	0.22 ***	----	Confirmed	0.42 ***	----	Confirmed
H2	Feedback → SATIS	0.47 ***	----	Confirmed	0.19 **	----	Confirmed
H3	Assessment → SATIS	0.42 ***	----	Confirmed	0.23 ***	----	Confirmed
H4	Participation and collaboration → SATIS	0.44 ***	----	Confirmed	0.27 ***	----	Confirmed
H5	Support and Motivation → SATIS	0.39 ***	----	Confirmed	0.25 ***	----	Confirmed
H6	Access to information and resources → SATIS	0.21 ***	----	Confirmed	0.41 ***	---	Confirmed
Student Satisfaction (SATIS)			0.83			0.65	

Before COVID-19 Model: “χ^2^ (399, N = 500) = 1087.071, *p* < 0.001, normed χ^2^ = 4.529, SRMR = 0.021, RMSEA = 0.029, CFI = 0.952, NFI = 0.935, TLI = 0.939, PNFI = 0.766, and PCFI = 0.782)”. Amid COVID-19 Model: “χ^2^ (399, N = 500) = 1964.277, *p* < 0.001, normed χ^2^ = 4.932, SRMR = 0.039, RMSEA = 0.048, CFI = 0.943, NFI = 0.927, TLI = 0.929, PNFI = 0.759, and PCFI = 0.773)”. *** *p* value less than 0.001; ** *p* value less than 0.0.

## Data Availability

Data is available upon request from researchers who meet the eligibility criteria. Kindly contact the first author privately through e-mail.
